# Regional Citrate Anticoagulation in CRRT: Successes, Pitfalls, and Sustainability from a Long-Term Single-Center Experience

**DOI:** 10.3390/jcm15051807

**Published:** 2026-02-27

**Authors:** Emanuele De Simone, Annalisa Guarino, Marco Pozzato, Dario Roccatello, Savino Sciascia, Roberta Fenoglio

**Affiliations:** 1 Center of Research of Immunopathology and Rare Diseases, Coordinating Center of Piemonte and Valle d’Aosta Network for Rare Diseases, and SCDU Nephrology and Dialysis, 10154 Turin, Italy; annalisaguarino1994@gmail.com (A.G.); marco.pozzato1@gmail.com (M.P.); dario.roccatello@unito.it (D.R.); savino.sciascia@unito.it (S.S.); roberta.fenoglio@unito.it (R.F.); 2 Department of Clinical and Biological Sciences, University of Turin, 10124 Torino, Italy

**Keywords:** regional citrate anticoagulation, continuous renal replacement therapy, circuit lifespan, environmental sustainability, Green Nephrology

## Abstract

**Background:** Regional citrate anticoagulation (RCA) is the recommended first-line strategy for continuous renal replacement therapy (CRRT), yet global implementation remains inconsistent. We reviewed our extensive single-center experience to provide a real-life evaluation of RCA feasibility, limitations, and environmental sustainability. **Methods**: We retrospectively analyzed RCA treatments performed in our center from 2012 to 2022. Technical performance was assessed by circuit patency and failure timing. Environmental impact was evaluated by simulating filter consumption and hazardous waste production compared to a projected strategy using unfractionated heparin (UFH). **Results**: RCA utilization grew progressively, reaching 98% of all treatments. The technique showed an 80.2% success rate and a robust safety profile, demonstrating excellent feasibility and efficacy in clinical practice. However, among circuits intended for 72 h survival, over one-third failed prematurely; segmented regression confirmed a critical high-risk period within the first 24 h, with a significantly higher rate of circuit loss compared to the subsequent period (*p* < 0.05). In our simulation, RCA adoption saved over 1000 filters, preventing the emission of approximately 12 tons of CO_2_ equivalent. **Conclusions**: RCA is a safe, effective, and environmentally appealing method of CRRT. However, significant room for improvement remains; refining the technique to increase circuit success rates is essential to optimize clinical efficiency and further minimize the ecological footprint of acute renal care.

## 1. Introduction

Regional citrate anticoagulation (RCA) is currently recognized as the gold standard for continuous renal replacement therapy (CRRT). Major clinical guidelines, including the Kidney Disease: Improving Global Outcomes [1], recommend RCA as the first-line anticoagulation strategy due to its superior circuit patency and reduced bleeding risk compared to systemic heparinization. Numerous trials and meta-analyses [2,3] have confirmed that RCA significantly extends filter life and improves the overall efficiency of the treatment by minimizing technical downtime.

Despite these established advantages, the global adoption of RCA remains inconsistent. Many centers are still hesitant to implement citrate-based protocols as a standard of care, primarily due to concerns regarding potential metabolic complications—such as citrate accumulation and acid-base imbalances—and the perceived complexity of the monitoring required. The translation of clinical guidelines into routine practice is surprisingly low in many geographical areas. Consequently, there remains a significant gap between evidence-based recommendations and routine “real-life” bedside practice [4].

While safety and efficacy have been widely debated, less attention has been paid to the broader impact of RCA on healthcare sustainability. In the modern era, the ecological footprint of medical procedures has become a primary concern. The concept of “Green Nephrology” [5] emphasizes the need to reduce hazardous waste and resource consumption. In this context, prolonging CRRT circuit lifespan is not only a clinical goal, but also a potential strategy to reduce the environmental impact of acute renal care.

The aim of this study was to review our extensive single-center experience to evaluate the feasibility of RCA in a real-world setting, focusing on clinical success rates, degree of implementation, and incidence of adverse events. We also sought to provide real-world evidence supporting the systematic implementation of RCA, while considering its broader implications for clinical and environmental management. Finally, we investigated the potential environmental implications of this approach by simulating a scenario based on a transition to unfractionated heparin (UFH), estimating the additional number of disposable filters required and the consequent increase in carbon footprint within the intensive care setting.

## 2. Materials and Methods

The study was conducted within an academic Nephrology Unit. CRRT with regional citrate anticoagulation was prescribed and managed by nephrologists and delivered across three critical care settings within the same hospital: a single general intensive care unit, an emergency medicine unit, and a cardiac intensive care unit, caring for a mixed population of medical and surgical critically ill patients.

### 2.1. CRRT Treatment Protocol and Technical Setting

For all treatments, the maximum intended duration for a single set (filter and circuit) was set at 72 h, in accordance with the manufacturer’s guidelines. The dialysis dose prescription was defined in collaboration with the consultant nephrologist, following an internal protocol based on body weight, aiming to deliver a target effluent dose between 20 and 25 mL/kg/h.

Over the analyzed decade (2012–2022), the following CRRT platforms were utilized: Prismaflex and PrisMax (Baxter, Deerfield, IL, USA), Amplya and Lynda (Bellco/Mozarc, Mirandola, Italy). The membranes employed were ST150 (Baxter), an acrylonitrile and sodium methallylsulfonate copolymer (AN69-ST) membrane surface-treated with polyethyleneimine (PEI) and the Clearum HS Series (Bellco), a high-flux polyethersulfone (PES) dialyzers with polyvinylpyrrolidone (PVP) additives.

The central venous catheters employed were made of polyurethane or silicone, had an 11.5-French diameter, and varied in length according to the insertion site and patient size (range 16–30 cm).

### 2.2. Regional Citrate Anticoagulation (RCA) Protocol

RCA was implemented via a pre-blood pump infusion of sodium citrate, targeting a citrate delivery of 3 mmol/L relative to blood flow in the extracorporeal circuit.

**Figure 1:**
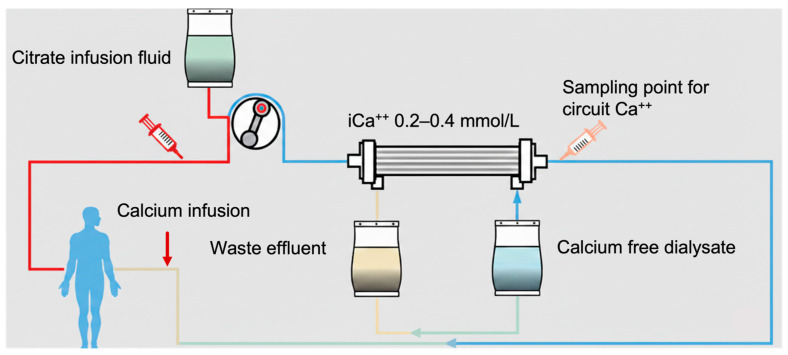
Schematic representation of the CRRT circuit employing regional citrate anticoagulation. Sodium citrate is infused pre-filter to chelate iCa^++^, maintaining circuit levels between 0.2 and 0.4 mmol/L to inhibit coagulation. Systemic iCa^++^ homeostasis is subsequently restored via calcium infusion into the venous return line.

Citrate neutralization was achieved by a return-line infusion of 10% calcium chloride (680 mmol/L CaCl_2_), adjusted to maintain systemic ionized calcium levels between 1.0 and 1.2 mmol/L. Post-filter ionized calcium was monitored to assess the anticoagulation (Figure 1). Replacement fluids consisted of pre-dilution solutions containing citrate at a fixed concentration of 18 mmol/L (Prismocitrate 18/0, Regiocit), with flow rates adjusted according to blood flow.

Between 2012 and 2014, Prismocitrate 10/2 was utilized. Regarding the dialysate, a calcium-free solution (Prism0cal) was consistently employed during all RCA treatments.

Metabolic monitoring during RCA was performed at defined intervals: at 30 min, 1 h, and 2 h after treatment initiation, and every 6 h thereafter. Total calcium was measured every 24 h to calculate the Total Calcium/Ionized Calcium (tCa/iCa) ratio. A safety cut-off of less than 2.5 was used as an early indicator of citrate accumulation (citrate gap).

### 2.3. Data Collection

Data were retrospectively collected from paper-based prescription forms and nursing monitoring logs. These included:

Machine Parameters: Mean values per treatment for blood flow (Qb), pre-infusion rate, post-infusion rate, and dialysate flow rate.

Pressure Monitoring: Mean values per treatment for access pressures (arterial and venous) and transmembrane pressure (TMP).

Clinical and Operational Data: Type of anticoagulant used, hospital unit, vascular access site (femoral or jugular central venous catheter), and primary diagnosis at admission.

Treatment Interruptions: Documentation of technical and non-technical interruptions (clinical or logistical reasons) and specific episodes of bleeding leading to treatment suspension.

### 2.4. Study Endpoints

The technical performance of RCA was evaluated by calculating the percentage of treatments completed as prescribed and the mean circuit patency in hours. Circuit thrombosis was defined as premature circuit failure requiring interruption of CRRT treatment and circuit replacement due to clotting. We analyzed the cumulative proportion of circuits lost due to clotting at intervals of 3, 6, 12, 24, 48 h. A specific sub-analysis was performed on treatments intended for 72 h patency, excluding ‘clinical censoring’ (e.g., patient death, renal recovery, or surgical necessity).

RCA safety was assessed by analyzing metabolic imbalances and citrate accumulation (citrate gap). Specific safety evaluations were performed on two high-risk subgroups: patients with metformin-associated lactic acidosis (MALA) and those with impaired liver function. The latter was defined by the presence of at least one baseline criterion: AST/ALT >3× upper limit of normal, total bilirubin > 5 mg/dL, INR > 1.5, or a known diagnosis of chronic liver disease/cirrhosis.

### 2.5. Statistical Analysis

Continuous variables were tested for normal distribution using visual inspection of histograms and Q–Q plots and are presented as mean ± standard deviation (SD) or median (interquartile range, IQR) as appropriate. Categorical variables are reported as absolute frequencies and percentages.

Comparisons between groups were performed using the independent-samples Student’s *t*-test or Mann–Whitney U test for continuous variables, depending on distributional assumptions. Categorical variables were compared using the χ^2^ test or Fisher’s exact test when appropriate. All tests were two-sided, and a *p*-value < 0.05 was considered statistically significant. Circuit survival was analyzed using two complementary approaches. First, descriptive time-to-event analysis was performed by calculating cumulative proportions of circuits lost to clotting at predefined time intervals (3, 6, 12, 24, 48, and 72 h). Treatments discontinued for non-clotting reasons (e.g., patient death, renal recovery, planned discontinuation, or vascular access failure) were considered clinically censored in the 72 h survival sub-analysis. Second, to formally evaluate the non-linear temporal pattern of cumulative circuit loss, we modelled the cumulative percentage of clotting-related failures using piecewise (segmented) linear regression. A prespecified knot was placed at 24 h based on visual inspection of the cumulative incidence curve and clinical plausibility of an early high-risk phase. Slopes before and after the knot were estimated using ordinary least squares regression with robust standard errors. The difference between slopes was assessed using a Wald test for interaction. As a sensitivity analysis, segmented regression with an unknown breakpoint was performed using maximum likelihood estimation to identify the optimal inflection point. The presence of a statistically significant change in slope was tested using the Davies test. This approach allowed formal confirmation of a structural change in the failure rate over time, supporting the identification of a distinct early high-risk period. To compare circuit lifespan between anticoagulation strategies (RCA vs. UFH), mean circuit duration was compared using an independent-samples *t*-test. The absolute difference in mean circuit survival (hours) was used as the basis for the environmental impact simulation. For the environmental sustainability analysis, total treatment hours delivered with RCA were divided by the observed mean circuit duration for each anticoagulation strategy to estimate the total number of circuits required under each scenario. The difference in estimated circuit consumption between RCA and a hypothetical UFH-only strategy was multiplied by the Life Cycle Assessment (LCA)-derived carbon footprint per circuit to estimate avoided CO_2_-equivalent emissions. No inferential statistical testing was applied to the simulation analysis, as it was based on deterministic modeling using observed cohort-level means.

All statistical analyses were performed using IBM SPSS Statistics (Version 26.0, IBM Corp., Armonk, NY, USA). Segmented regression and breakpoint analyses were conducted using validated add-on procedures for structural change modeling.

### 2.6. Environmental Impact Analysis

To explore the potential environmental implications of circuit longevity, we performed a simulation-based analysis comparing the observed circuit lifespan under regional citrate anticoagulation with that observed during unfractionated heparin use in the same center. The difference in mean circuit duration between the two anticoagulation strategies was used to estimate the number of additional CRRT circuits that would have been required under a hypothetical heparin-only strategy over the total treatment hours delivered. The environmental impact was estimated using Life Cycle Assessment (LCA) data provided by the manufacturer for the Prismaflex system.

Generative artificial intelligence tools were used to assist with language editing of the manuscript.

## 3. Results

### 3.1. Cohort Characteristics and Study Population

**Figure 2:**
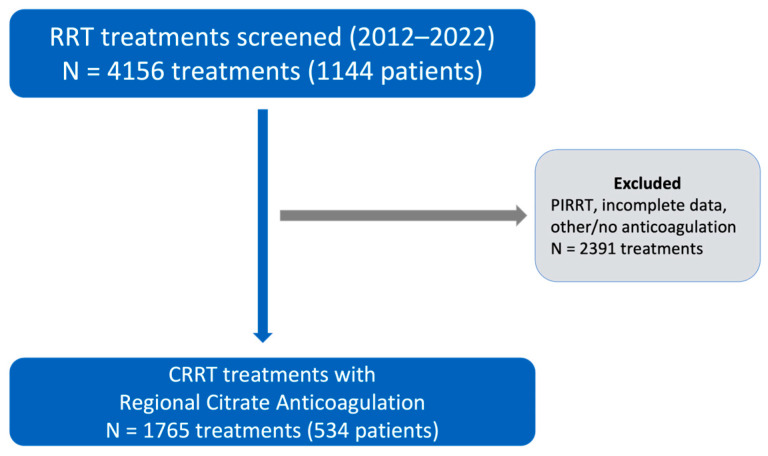
Flowchart illustrating the selection of CRRT treatments from all extracorporeal renal replacement therapy (RRT) treatments screened.

We retrospectively analyzed a total of 4156 treatments performed on 1144 patients. After excluding treatments categorized as Prolonged Intermittent Renal Replacement Therapy (PIRRT), sessions with incomplete data and treatments with other or no anticoagulation, a total of 1765 CRRT treatments performed with RCA were included in the analysis (Figure 2). All treatments were performed using diluted citrate in continuous venovenous hemodiafiltration mode with simultaneous pre- and post-dilution.

The mean age of the RCA cohort was 68.2 ± 14 years, with a predominance of male patients (*n* = 346, 64.8%). The average number of RCA treatments per patient was 3.3 (1–9). The overall mortality rate within this cohort was 39.7% (*n* = 212).

**Figure 3:**
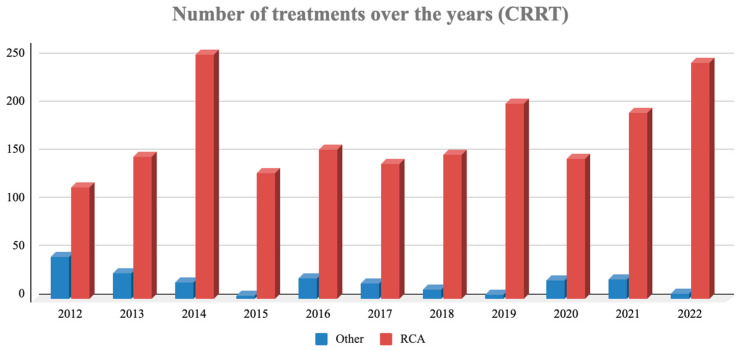
CRRT anticoagulation trends (2012–2022). Annual distribution of CRRT treatments. Red bars: regional citrate anticoagulation (RCA); blue bars: other modalities (mainly UFH).

As shown in Figure 3, the implementation of RCA grew progressively and significantly over the decade, starting from 72.5% of total CRRT treatments in 2012 (116/160) and reaching 98.0% by 2022 (245/250).

The primary indications for CRRT with citrate anticoagulation were sepsis (29.4%, *n* = 157) and post-surgical complications (13.3%, *n* = 71). Fluid overload was the main driver for 12.2% of the cases (*n* = 65), while the “Sepsis combined” category—representing sepsis associated with other major acute comorbidities—accounted for an additional 8.2% of patients.

Femoral venous access was utilized in the vast majority of RCA treatments (*n* = 1530, 86.7%).

The operational settings and machine parameters for the RCA treatments are summarized in Table 1.

### 3.2. Circuit Performance and Survival Analysis

**Figure 4:**
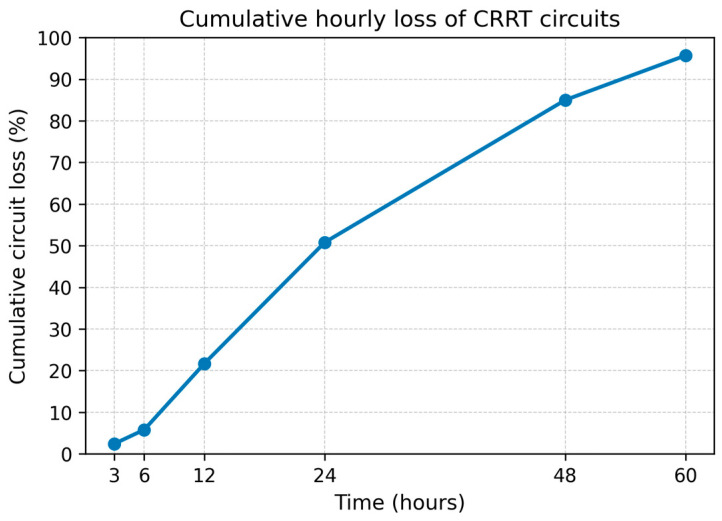
Cumulative hourly loss of CRRT circuits due to clotting. The figure shows the cumulative percentage of CRRT circuits lost because of clotting over time, expressed as a function of treatment duration. Circuit loss increased rapidly during the early phase of treatment, with approximately half of all clotting events occurring within the first 24 h, followed by a slower rate of loss between 24 and 72 h.

In the RCA group, 1415 treatments (80.2%) were completed as prescribed, with a mean circuit life of 45 ± 21 h. Analysis of premature RCA terminations (*n* = 350) identified technical issues (*n* = 3), vascular access failure (*n* = 20), and clotting (*n* = 327). For circuits that failed due to clotting, the mean survival was 26.5 ± 16 h. After excluding non-clotting related interruptions (such as clinical censoring or access failure), the probability of a circuit reaching the 72 h physiological limit was 63.3% (565/892). The cumulative incidence curve demonstrated a clear time-dependent pattern of CRRT circuit loss, with a steeper increase during the early treatment phase. The estimated slope of cumulative circuit loss between 3 and 24 h was 2.3% per hour (95% CI 1.9–2.7), compared with 0.9% per hour (95% CI 0.6–1.2) between 24 and 72 h. The difference in slopes was statistically significant (Wald test, *p* < 0.001), indicating a substantially higher rate of circuit failure during the first 24 h of CRRT. In sensitivity analyses, segmented regression identified a significant change in slope occurring between 22 and 26 h, which was confirmed by the Davies test (*p* = 0.002), supporting the robustness of a distinct early high-risk period for circuit loss (Table 2 and Figure 4).

### 3.3. Safety and Metabolic Tolerance

Citrate accumulation (Total Calcium/Ionized Calcium ratio > 2.5) was observed in only 3.5% of RCA treatments. Metabolic alkalosis was rare (0.5%), while metabolic acidosis (21.9%) was rarely associated with citrate accumulation (only 0.9% of total treatments). Only one treatment (0.1%) required definitive discontinuation due to citrate-related metabolic issues.

In patients with impaired liver function (*n* = 121), the accumulation rate was slightly higher (4.7% vs. 2.9% in non-cirrhotic patients), but this difference did not reach statistical significance (*p* = 0.06). In patients with Metformin-Associated Lactic Acidosis (MALA), the method proved feasible with a mean tCa/iCa ratio of 2.1 after 24 h.

### 3.4. Environmental Sustainability

To evaluate the environmental implications of our anticoagulation strategy, we performed a simulation comparing the observed RCA circuit survival with the performance of unfractionated heparin (UFH) utilized during the same study period. In our cohort, the mean circuit life for UFH was 28 ± 17 h, representing a significant difference of 17 h compared to the RCA group.

The sustainability analysis was based on the specific carbon footprint of the Prismaflex system, which was utilized for 1744 (98.8%) of the total RCA treatments. By modeling the 17 h difference in circuit survival over the total treatment hours provided, we estimated that a hypothetical exclusive use of UFH would have required the consumption of an additional 1071 filters and disposable sets. According to the Life Cycle Assessment (LCA) data provided by the manufacturer for the Prismaflex sets, each complete circuit has a carbon footprint of 11.2 kg of CO_2_ equivalent [6]. Consequently, the systematic adoption of RCA prevented the emission of approximately 12 tons of CO_2_ equivalent. 

## 4. Discussion

The retrospective analysis of our ten-year experience provides a reasoned evaluation of the progress made at our Center since the introduction of regional citrate anticoagulation (RCA). Following the 2012 KDIGO guidelines, which established RCA as the preferred method for CRRT, we observed a progressive increase in its utilization, reaching 98% of all treatments by 2022. This trend indirectly confirms the feasibility and sustainability of the technique.

Despite strong international recommendations, the literature highlights that the adoption of RCA remains heterogeneous and often lower than expected, hindered by concerns regarding metabolic complexity. In fact, recent data indicate that RCA is not yet a universal global standard. In the United States, its use remains surprisingly limited as recent analyses suggest only a minority of patients receive RCA during CRRT, with UFH used in most cases [4]. In Europe, although more widespread, adoption is inconsistent. In the United Kingdom, RCA is used in over 50% of intensive care units (ICUs) [7], while in Italy, a joint survey reported its use as a first-line choice in 76% of centers [8]. Our data showing 98% utilization represents one of the highest reported levels of adherence to international guidelines. Conversely, in Asia (China, Japan, and Republic of Korea), UFH remains the prevailing method despite increasing integration of RCA into referral center protocols [7]. While implementation costs are often cited as barriers, studies in resource-limited settings have demonstrated that RCA can be implemented successfully and safely through rigorous protocols [9].

Our results confirm the high technical performance of regional citrate anticoagulation (RCA), aligning with major clinical trials and meta-analyses. In our cohort, the mean circuit patency was 45.0 ± 21 h. This result is consistent with the findings of the RICH trial, which reported an average filter life of 47.2 h [2]. Similar results were reported by Gattas et al. [10], with a median circuit survival of 39.2 h. Broader systematic reviews further confirm this trend [11,12,13,14], showing a global mean duration of approximately 36.7 h for RCA-managed circuits [15], with a reported 12 h gain in mean circuit life for RCA compared to unfractionated heparin (UFH) [7], a finding similar to the 17 h difference observed in our cohort. Furthermore, our technical success rate—defined as treatments completed without premature clotting—reached 80.2%. This performance aligns with the literature, where clotting-related failure rates for citrate typically range from 13% to 21% [3,16,17].

Regarding safety, the method showed excellent tolerability. Metabolic complications requiring discontinuation occurred in only a single case (0.1%), involving a patient with liver dysfunction. Metabolic alkalosis was rare (0.5%), a figure notably lower than the 2.4% reported in the RICH trial [2]. We believe this low incidence is correlated with the evolution of citrate-specific solutions, such as those with reduced bicarbonate or enriched with phosphates and magnesium. The risk profile remained acceptable even in historically critical subgroups, such as patients with impaired liver function and those with Metformin-Associated Lactic Acidosis (MALA), for whom we have previously published specific evidence [18]. While RCA was generally avoided in cases of fulminant liver failure or severe hyperlactatemia, the 98% utilization rate suggests that exclusion criteria were applied very selectively, indicating that “prohibitive” cases for citrate are rare in real-world clinical practice. Safety is further supported by the absence of hemorrhagic events in the RCA group, compared to five UFH treatments suspended due to active bleeding.

A more critical aspect of our data concerns circuit survival. Despite the clear superiority of regional citrate anticoagulation (RCA) to heparin, approximately 37% of circuits did not reach the 72 h physiological limit, with 50% of these failures occurring within the first 24 h. While our results surpass the 72 h survival rates reported in the RICH trial and by Gattas et al. [2,10], they indicate that at least one-third of circuits are lost prematurely during long-duration treatments. Our analysis differs from the standard time-to-event survival analysis of clinical trials. In fact, we focused exclusively on filters intended for 72 h use that were lost specifically due to clotting. Using segmented regression analysis, we identified a distinct early high-risk period, with a significant change in the failure slope occurring between 22 and 26 h. This non-linear pattern was confirmed by the Davies test (*p* = 0.002), which identified a significant “knot” at approximately 24 h. The risk of circuit loss was significantly higher during this initial phase (2.3% per hour) compared to the subsequent period (0.9% per hour; Wald test *p* < 0.001). This approach reveals that once the 24 h threshold is passed, the probability of circuit survival increases substantially. While our protocol included strict post-filter ionized calcium monitoring, recent evidence suggests that borderline calcium levels are unlikely to be the primary driver of premature failure [19]. Therefore, it is probable that non-calcium-dependent factors are the primary drivers of early loss. Investigating these mechanisms remains challenging because standard coagulation tests are inadequate for monitoring circuit patency during RCA.

Beyond clinical outcomes, premature circuit clotting has significant implications for environmental sustainability. In the era of “Green Nephrology,” reducing the consumption of disposables is a key strategic goal [20,21]. Our analysis demonstrates that initiating a CRRT session with regional citrate anticoagulation (RCA) provides a nearly five-fold higher probability of reaching the 72 h physiological target compared to heparin-based strategies. Consequently, we calculated that managing our cohort exclusively with contemporary unfractionated heparin (UFH) protocols would have required an additional 1071 filters and disposable sets. This impact extends far beyond the filters themselves. In fact, every circuit change involves substantial amounts of packaging, tubing, and auxiliary disposables, leading to a significant increase in the production of hazardous medical waste. While specific data for CRRT are limited, previous studies in traditional hemodialysis have estimated that each session generates approximately 2.5 kg of plastic and hazardous waste, providing a reliable benchmark for the environmental burden of extracorporeal circuits [5]. Furthermore, our simulation—based on the Life Cycle Assessment (LCA) of the Prismaflex system—indicates that the increased circuit survival achieved with RCA prevented the emission of 11.99 tons of CO_2_ equivalent (Baxter International Inc., Deerfield, IL, USA, 2021). Beyond the reduction in waste, recent publications indicate the citrate represents a more ecologically efficient alternative to heparin [22]. The industrial production of heparin is energy-intensive and involves complex animal-based supply chains and logistics, whereas the synthesis of citrate is more chemically and ecologically efficient.

Our study has limitations due to the intrinsic nature of the retrospective, single-center analysis; however, its close alignment with RCT data provides significant robustness to these real-life, unselected data.

In conclusion, RCA should be considered the method of choice for the vast majority of CRRT treatments, as it proves to be safe, feasible, and the most environmentally sustainable option currently available. Nevertheless, room for improvement exists, as early clotting continues to affect a relevant fraction of sessions, jeopardizing resource consumption and increasing the environmental burden of intensive care.

## Figures and Tables

**Table 1:** Population characteristics and CRRT operational parameters. Data are presented as mean ± SD or *n* (%). Qb: blood flow; TMP: transmembrane pressure.

Category	Parameter	Value
Demographics	Total Patients (*n*°)	534
	Gender (Male/Female)	394 (64.8%)/188 (35.2%)
	Mean Age (years)	68.2 ± 14
	Mean Treatments per Patient	3.3 (1–9)
Vascular Access	Right Femoral	993
	Left Femoral	532
	Right Jugular	153
	Left Jugular	82
	Predominant Site (Femoral)	86.7%
Primary Diagnosis	Sepsis (Total/Combined)	36.1%
	Post-surgical	14.4%
	Fluid Overload	11.8%
	Exogenous Intoxication	3.7%
	Other	34.0%
Operational Data	Blood Flow (Q_b_)	123 ± 19 mL/min
	Pre-infusion Rate	1359 ± 520 mL/h
	Post-infusion Rate	486 ± 177 mL/h
	Dialysate Flow Rate	1225 ± 209 mL/h
	Mean TMP	80 ± 23 mmHg
	Mean Access Pressure	76 ± 30 mmHg
	Mean Return Pressure	69 ± 31 mmHg

**Table 2:** RCA technical performance and circuit failure analysis. Summary of technical outcomes across 1765 regional citrate anticoagulation (RCA) treatments. Data are presented as *n*, mean ± SD, or percentages. * The 72 h success rate refers specifically to circuits reaching the physiological limit without clotting; clotting rates and cumulative timings are calculated based on circuits lost prematurely to filter coagulation.

Category	Parameter	RCA Value
Global Performance	Total RCA Treatments Analyzed (*n*)	1765
	Mean Circuit Life (hours)	45 ± 21
	Treatments Completed as Prescribed	80.2%
	72 h Success Rate *	63.3%
Technical Failures	72 h Clotting Rate	36.7%
	Total Circuits Lost to Clotting (*n*)	327
	Mean Survival of Clotted Circuits (hours)	26.5 ± 16
Cumulative Failure Timing	Clotted < 3 h	2.4%
	Clotted < 6 h	5.8%
	Clotted < 12 h	21.7%
	Clotted < 24 h	50.8%
	Clotted < 48 h	85.0%
Other Interruption Reasons	Vascular Access Failure (*n*)	20
	Mechanical/Technical Issues (*n*)	3
	Citrate-related Discontinuation (*n*)	1 (0.1%)

## Data Availability

Data are available upon reasonable request.

## Abbreviations

The following abbreviations are used in this manuscript:

AKI	Acute Kidney Injury
ALT	Alanine Aminotransferase
AST	Aspartate Aminotransferase
CRRT	Continuous Renal Replacement Therapy
HIT	Heparin-Induced Thrombocytopenia
INR	International Normalized Ratio
IU	International Units
KDIGO	Kidney Disease: Improving Global Outcomes
MALA	Metformin-Associated Lactic Acidosis
PEI	Polyethyleneimine
PES	Polyethersulfone
PIRRT	Prolonged Intermittent Renal Replacement Therapy
PVP	Polyvinylpyrrolidone
RCA	Regional Citrate Anticoagulation
SD	Standard Deviation
TMP	Transmembrane Pressure
UFH	Unfractionated Heparin
